# Subpopulations of Projection Neurons in the Olfactory Bulb

**DOI:** 10.3389/fncir.2020.561822

**Published:** 2020-08-28

**Authors:** Fumiaki Imamura, Ayako Ito, Brandon J. LaFever

**Affiliations:** Department of Pharmacology, Penn State College of Medicine, Hershey, PA, United States

**Keywords:** olfactory bulb, projection neurons, heterogeneity, parallel pathways, mitral cell, tufted cell

## Abstract

Generation of neuronal diversity is a biological strategy widely used in the brain to process complex information. The olfactory bulb is the first relay station of olfactory information in the vertebrate central nervous system. In the olfactory bulb, axons of the olfactory sensory neurons form synapses with dendrites of projection neurons that transmit the olfactory information to the olfactory cortex. Historically, the olfactory bulb projection neurons have been classified into two populations, mitral cells and tufted cells. The somata of these cells are distinctly segregated within the layers of the olfactory bulb; the mitral cells are located in the mitral cell layer while the tufted cells are found in the external plexiform layer. Although mitral and tufted cells share many morphological, biophysical, and molecular characteristics, they differ in soma size, projection patterns of their dendrites and axons, and odor responses. In addition, tufted cells are further subclassified based on the relative depth of their somata location in the external plexiform layer. Evidence suggests that different types of tufted cells have distinct cellular properties and play different roles in olfactory information processing. Therefore, mitral and different types of tufted cells are considered as starting points for parallel pathways of olfactory information processing in the brain. Moreover, recent studies suggest that mitral cells also consist of heterogeneous subpopulations with different cellular properties despite the fact that the mitral cell layer is a single-cell layer. In this review, we first compare the morphology of projection neurons in the olfactory bulb of different vertebrate species. Next, we explore the similarities and differences among subpopulations of projection neurons in the rodent olfactory bulb. We also discuss the timing of neurogenesis as a factor for the generation of projection neuron heterogeneity in the olfactory bulb. Knowledge about the subpopulations of olfactory bulb projection neurons will contribute to a better understanding of the complex olfactory information processing in higher brain regions.

## Introduction

Our ability to perceive the world through our senses begins with different sensory organs and results in distinct brain regions processing this information. However, even a single sensory system must process multiple aspects of the sensory modality in order to generate a meaningful sensory experience. For example, we can acquire various types of object information, such as size and shape, color, brightness, location, and motion, through the visual system (Wandell, 1995). A strategy to process multiple visual submodalities is to enhance the functional diversity of the neuronal circuit by expanding the neuronal heterogeneity in the retina (MacNeil and Masland, 1998; Masland, 2012; Baden et al., 2016; Yan et al., 2020). In the primate visual system, strong evidence suggests that the color and motion of an object are differentially processed through parallel pathways (Nassi and Callaway, 2009). These parallel pathways are formed by different types of retinal ganglion cells (RGCs) projecting their axons to different layers of the lateral geniculate nucleus of the thalamus and subsequently different regions of the cortex (Nassi and Callaway, 2009; Schiller, 2010; Seabrook et al., 2017). Thus far, at least 20 different RGC subtypes have been identified based on their different morphological, physiological, and molecular properties in the primate retina (Kolb et al., 1992; Peng et al., 2019). Recent studies expanded the classification of the cells into 40 RGC subtypes in the mouse retina (Sanes and Masland, 2015; Baden et al., 2016; Rheaume et al., 2018).

In terms of evolution, the olfactory system is one of the oldest senses to monitor the outside world. It is said that there are more than 3,000 chemicals that can be detected by our chemosensory system as fragrances (Arctander, 1969), and the mixture of these chemicals produces different odor qualities and behavioral responses (Kay et al., 2005; Saraiva et al., 2016). Unlike the visual system that receives photons with 3–6 types of photoreceptor cells (rod cells and 2–5 types of cone cells) (Baden et al., 2020), the vertebrate olfactory system takes a unique strategy to discriminate these odor qualities by establishing a large repertoire of odorant receptors expressed by olfactory sensory neurons (OSNs) (Buck and Axel, 1991; Zhang and Firestein, 2002; Godfrey et al., 2004; Malnic et al., 2004). However, each OSN expresses only a single type of odorant receptor (Chess et al., 1994; Serizawa et al., 2003; Monahan and Lomvardas, 2015). All OSNs project axons to the olfactory bulb (OB), a structure that is found in all vertebrate animals and the first relay station for the olfactory information in the central nervous system (Mombaerts et al., 1996; Northcutt, 2002). The OSNs expressing the same type of odorant receptor converge their axons usually into 2-3 spherical structures known as glomeruli (Mombaerts et al., 1996). The convergence of OSN axons forms a glomerular map, or odorant receptor map, at the surface of the OB (Mori et al., 1999; Mori and Sakano, 2011). The information from the glomerular map is transmitted to the olfactory cortex through the axons of OB projection neurons (Ghosh et al., 2011; Sosulski et al., 2011; Igarashi et al., 2012; Hirata et al., 2019).

A single odor or an odor mixture activates a distinct combination of OSNs and glomeruli (Malnic et al., 1999; Mori et al., 2006; Johnson and Leon, 2007; Fletcher, 2011). Therefore, processing the glomerular activation pattern on the map is the first step to identify the odors. In addition, the olfactory system processes different aspects of smell sensation; not only the odor quality, but also the odor intensity, pleasantness, and location of the source (Thomas-Danguin et al., 2014). Different concentrations of the same odor activate different numbers of glomeruli and change the temporal profiles of OB projection neuron responses (Johnson and Leon, 2000; Spors and Grinvald, 2002; Gautam et al., 2014; Bolding and Franks, 2018). Specific domains in the glomerular map are responsible for mediating particular odor-induced behaviors in rodents (Bear et al., 2016). For example, signals from the dorsomedial glomeruli are responsible for the innate fear responses caused by predator odors in mice, which is likely controlled by the central amygdala (Kobayakawa et al., 2007; Dewan et al., 2013; Root et al., 2014; Isosaka et al., 2015; Kondoh et al., 2016). In contrast, a subset of ventral glomeruli are targeted by TRPM5 expressing OSNs that are responsible for semiochemical processing, and these glomeruli are innervated by a population of mitral cells projecting to the medial amygdala (Lin et al., 2007; Thompson et al., 2012; Lemons et al., 2017). Mounting evidence demonstrates the importance of OB organization in information processing.

Here, we focus on the subtypes of OB projection neurons. Accumulating evidence shows that OB projection neurons can be subdivided into several subpopulations with different morphological and physiological properties. This suggests that different subpopulations of OB projection neurons may be involved in processing different aspects of smell via parallel pathways. We begin by summarizing the OB projections neurons found in lower vertebrate species to analyze their diversity from an evolutionary perspective, focusing primarily on their morphological properties. Then, we describe the similarities and differences among the subpopulation of projection neurons in the rodent OB. Lastly, we discuss the timing of neurogenesis as a factor for generating heterogeneity of projection neurons in the OB. The authors apologize to those whose work was not included here due to space limitations.

## Basic Neural Circuitry of the Mammalian Olfactory System

**FIGURE 1:**
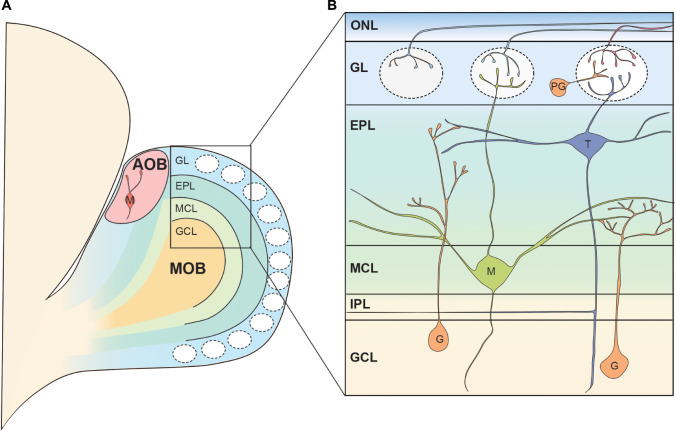
Basic neural circuit in the rodent olfactory bulb. **(A)** The olfactory bulb (OB) is formed at the most anterior portion of the telencephalon in the rodent brain. The accessory olfactory bulb (AOB) receiving the pheromonal information from the vomeronasal organ is located at the posterodorsal OB. Mitral cells in the AOB have multiple apical dendrites projecting to multiple glomeruli. The rest of the OB is called the main OB (MOB) and is innervated by olfactory sensory neurons (OSNs). The MOB consists of multiple concentric layers. **(B)** The OSN axons tangentially run at the surface of the MOB within the olfactory nerve layer (ONL), before entering the glomeruli. Mitral (M) and tufted cell (T) somata are located in the mitral cell layer (MCL) and external plexiform layer (EPL), respectively, and project their primary dendrites to a single glomerulus. In the glomerulus, OSN axons form axodendritic synapses with mitral and tufted cells as well as periglomerular cells (PG). The secondary dendrites of mitral and tufted cells form dendrodendritic synapses with granule cells (G) in the EPL. Somata of periglomerular and granule cells are found in the glomerular layer (GL) and the granule cell layer (GCL), respectively. IPL, internal plexiform layer.

The mammalian main OB (MOB) has an onion-like layer structure consisting of various cellular populations segregated into individual layers. Figure 1 shows the structure of the rodent OB as an anatomical model for reference. Although we do not go deeper into detail in this review, there are many excellent reviews summarizing the cell types, synapses, and neuronal circuits found in the MOB (Shepherd et al., 2004; Wachowiak and Shipley, 2006; Figueres-Onate et al., 2014; Imai, 2014; Nagayama et al., 2014; Sakano, 2020). Briefly, the OSN axons run tangentially through the olfactory nerve layer (ONL) at the surface of the OB before entering the glomerular layer (GL) (Klenoff and Greer, 1998; Rodriguez-Gil et al., 2015). Here, the OSNs form axodendritic synapses with the OB projection neurons, known as mitral and tufted cells, as well as periglomerular interneurons (Pinching and Powell, 1971b; White, 1972). In addition, OB projection neurons and periglomerular interneurons form reciprocal dendrodendritic synapses within the GL (Pinching and Powell, 1971b; White, 1972). Both mitral and tufted cells are glutamatergic neurons, and they share morphological features such as a single primary dendrite projecting to a single glomerulus as well as the secondary dendrites extending within the external plexiform layer (EPL), a layer beneath the GL (Macrides and Schneider, 1982; Mori et al., 1983). Within the EPL, the secondary dendrites of mitral and tufted cells form dendrodendritic synapses with granule cells, another type of interneuron (Rall et al., 1966; Price and Powell, 1970b). The somata of most tufted cells are found in the EPL while the mitral cell somata are aligned below the EPL to form a thin layer called the mitral cell layer (MCL) (Schneider and Macrides, 1978). The granule cell layer (GCL) is located below the MCL and is the largest layer in the OB formed primarily by the somata of granule cells (Schneider and Macrides, 1978). There is another thin layer between the MCL and GCL known as the internal plexiform layer (IPL) which contains axon collaterals from tufted cells (Liu and Shipley, 1994).

In addition to the main olfactory system, rodents have a vomeronasal system, unlike humans, through which they process pheromonal signals with great sensitivity (Silva and Antunes, 2017). For further details on aspects of the organization of the neuronal circuits involved in the pheromonal signals, readers are referred to several excellent and comprehensive studies and reviews (Larriva-Sahd, 2008; Tirindelli et al., 2009; Yokosuka, 2012; Silva and Antunes, 2017; Holy, 2018; Mohrhardt et al., 2018). In the vomeronasal organ, pheromones are received by vomeronasal receptors expressed by vomeronasal sensory neurons, which project axons to the accessory olfactory bulb (AOB) located at the dorsoposterior region of the MOB (Belluscio et al., 1999; Rodriguez et al., 1999; Boschat et al., 2002; Del Punta et al., 2002). Similar to the MOB, there are glomeruli at the surface of the AOB, where the AOB mitral cells receive synaptic inputs from vomeronasal neurons (Barber et al., 1978). However, upon reaching the AOB, individual axons can divide to terminate in multiple glomeruli (Larriva-Sahd, 2008). Therefore, contrary to the MOB, axons of sensory neurons expressing a given receptor form multiple glomeruli in the AOB (Belluscio et al., 1999; Rodriguez et al., 1999). Somata of AOB mitral cells are scattered beneath the glomeruli and a single AOB mitral cell possesses multiple primary dendrites which innervate multiple glomeruli (Takami and Graziadei, 1991; Wagner et al., 2006; Yonekura and Yokoi, 2007; Larriva-Sahd, 2008). The AOB mitral cells extend secondary dendrites in the layer beneath the glomeruli where they form dendrodendritic synapses with AOB granule cells whose somata are localized at the deepest layer of the AOB (Yonekura and Yokoi, 2007; Larriva-Sahd, 2008). Although it is said that AOB mitral cells are the only type of projection neurons in the AOB, at least three types of AOB projection neurons with different somata shape, location, and dendritic morphology have been suggested to exist (Yonekura and Yokoi, 2007; Larriva-Sahd, 2008). In addition, a recent study showed that a subset of AOB projection neurons was intrinsically rhythmogenic (Gorin et al., 2016; Zylbertal et al., 2017). These results raise the possibility that AOB mitral cells also consist of highly heterogeneous subpopulations. The mitral cell axons exit the AOB in bundles and run through a layer between the somata of mitral cells and granule cells before converging on the lateral olfactory tract (LOT), ultimately transmitting the pheromonal information to higher brain regions (von Campenhausen and Mori, 2000; Larriva-Sahd, 2008).

## Morphology of OB Projection Neurons in Lower Vertebrates

### Fish

**FIGURE 2:**
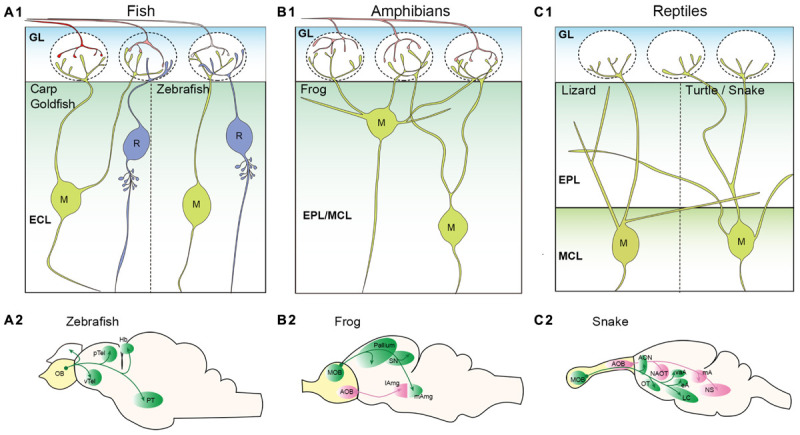
Projection neurons in the non-mammalian vertebrate olfactory bulb. **(A)** Schematic illustrations of projection neurons in the teleost fish olfactory bulb (OB). Mitral (M), and ruffed cells (R) are located in the external cell layer (ECL) **(A1)**. Most teleost fish including carp and goldfish have mitral cells with multiple apical dendrites projecting to multiple glomeruli. However, zebrafish mitral cells have only a single apical dendrite. No secondary dendrites are observed in the mitral cells. A morphological characteristic of ruffed cells is a membranous field surrounding the initial part of the axon. Axonal projection patterns from the zebrafish OB are shown in **(A2)** (Miyasaka et al., 2014). **(B)** Schematic illustrations of projection neurons in the amphibian OB. Somata of frog mitral cells (M) are located in the external plexiform layer (EPL) and mitral cell layer (MCL); these two layers are not clearly segregated **(B1)**. Mitral cells have multiple apical dendrites, and some have secondary dendrites that do not project to the glomeruli. Axonal projection pattern from the frog OB are shown in **(B2)** (Eisthen and Polese, 2010). **(C)** Schematic illustrations of projection neurons in the reptile OB. OSN axons are not depicted as it has not been clearly shown whether a single OSN of reptiles project unbranched axon to a single glomerulus. Somata of mitral cells (M) are found in the MCL that is clearly segregated from the EPL. A single primary dendrite is formed in lizard mitral cells, while multiple primary dendrites are found in turtle mitral cells **(C1)**. Both lizard and turtle mitral cells have clear secondary dendrites extending within the EPL. Axonal projection pattern from the snake OB are shown in **(C2)** (Eisthen and Polese, 2010; Ubeda-Banon et al., 2011). pTel and vTel, posterior and ventral telencephalon; Hb, habenula; PT, posterior tuberculum; SN, septal nucleus; lAmg and mAmg, lateral and medial amygdala; AON, anterior olfactory nucleus; OT, olfactory tubercle; LC, lateral cortex; NAOT, nucleus of accessory olfactory tract; vaA, eA, and mA, ventral anterior, external, and medial amygdala; and NS, nucleus sphericus.

The olfactory system of teleost fish sends the unbranched OSN axon to a single glomerulus (Weiss et al., 2020). The layer structure of the teleost fish OB is similar to that of the rodent OB, but a bit more simplistic (Kermen et al., 2013). In the fish OB, there are two types of glutamatergic projection neurons; mitral cells, a major projection neuron, and ruffed cells (Figure 2A1). There is no clear MCL, and both mitral cells and ruffed cells are located in the external cell layer that lies beneath the GL (Satou, 1990). Although they share the same name and have apical dendrites innervating glomeruli allowing them to receive input from OSN axons, the mitral cells of teleost fish are significantly different from those in the mammalian OB. Golgi staining and retrograde tracing of OB neurons in adult teleosts revealed that mitral cells do not extend secondary dendrites (Kosaka and Hama, 1982; Fuller et al., 2006). In most teleost fish, mitral cells possess multiple apical dendrites that project to multiple glomeruli, with the exception of zebrafish (Kosaka and Hama, 1982; Oka, 1983; Fujita et al., 1988; Satou, 1990; Fuller et al., 2006). The zebrafish mitral cells typically have only a single apical dendrite innervating a single glomerulus, and a smaller percentage of mitral cells have multiple apical dendrites that still innervate a single glomerulus (Fuller et al., 2006). In contrast to the mitral cells, the ruffed cells have a membranous field surrounding the initial part of the axon (Kosaka and Hama, 1979; Fuller and Byrd, 2005). A major difference from mitral cells is that ruffed cells do not receive direct input from OSN axons, but receive inhibitory synaptic inputs from interneurons activated by mitral cells (Kosaka, 1980; Satou, 1990). Therefore, the activity patterns of mitral and ruffed cells are contrasting in nature (Zippel, 1999; Zippel et al., 2000).

Mitral and ruffed cells project their axons to higher brain regions through either the medial olfactory tract (MOT) or LOT depending on the location of their somata (Fuller et al., 2006; Miyasaka et al., 2014). In the brain, mitral cell axons send their projections to five major regions; posterior and ventral telencephalon, posterior tuberculum, right habenula, and ipsilateral/contralateral OB (Eisthen and Polese, 2010) (Figure 2A2). Genetic labeling of zebrafish mitral cells at single-cell resolution revealed that (1) individual mitral cells can target multiple regions; the MOT and LOT do not determine the target regions, (2) mitral cells innervating the same glomerulus do not show the same axon trajectory and (3) the right habenula is innervated by mitral cells that convey the information from the medial glomeruli (Miyasaka et al., 2009, 2014).

Although there is no distinct AOB in the teleost OB, a report suggested that a specific glomerulus located at the mediodorsal OB, mdG2, may be functionally similar and serve as the accessory olfactory system in teleost fish (Biechl et al., 2017). The mdG2 receives inputs solely from crypt cells, a specific type of OSN activated by kin odor, and the mitral cells targeting the mdG2 send their axons to the intermediate ventral telencephalic nucleus (Ahuja et al., 2013; Biechl et al., 2016, 2017; Gerlach et al., 2019). It is hypothesized that this nucleus in teleost fish may be functionally analogous to the medial amygdala of mammals (Biechl et al., 2017). The evolutionary origin of the accessory olfactory system is an intriguing topic to aid in our understanding of the parallel pathways for olfactory information processing. The axonal projection patterns of the ruffed cells in the fish olfactory system is another research topic that remains to be elucidated.

### Amphibians

In contrast to the mammalian olfactory system, a single OSN of amphibians typically innervates multiple glomeruli (Gilbert et al., 2013; Hassenklover and Manzini, 2013). The OB of the frog has a layer structure similar to, but not as fully concentric as, that of the mammalian OB (Byrd and Burd, 1991; Scalia et al., 1991a; Kratskin et al., 2000). The projection neurons called mitral cells are scattered among the EPL and MCL (Kratskin et al., 2000). The basic morphology of the frog mitral cells resembles that of rodent mitral and tufted cells, except for their multiple (1–6) primary dendrites that innervate multiple glomeruli (Jiang and Holley, 1992) (Figure 2B1). Jiang and Holley (1992) further demonstrated that the mitral cells located superficially, close to the GL, extend their primary dendrites with a larger angle and targeted glomeruli across a wider field of range. The primary and secondary dendrites of the frog mitral cells are not clearly distinct in that some dendrites send several branches into the GL while the others remain in the EPL (Jiang and Holley, 1992). Mitral cells with similar morphology were also observed in the salamander OB (Herrick, 1924). A unique feature of amphibians is the process of metamorphosis in which species transform from an immature to mature state. This is an intriguing process with respect to olfactory system development and evolution. The studies of the OB morphology in the clawed frog showed that the basic structure of the mature OB was apparent in tadpoles (around larval stage 48/49) and remained constant throughout the late larval stage and into adulthood, with only the size increasing (Byrd and Burd, 1991; Nezlin and Schild, 2000). The majority of MOB projection neurons in both frog and salamander tadpoles (stages 51–56) had more than one glomerular tuft (up to 4) innervating different glomeruli (Herrick, 1924; Nezlin et al., 2003).

As shown in Figure 2B2, OB projection neurons project to the dorsal, lateral, and medial pallium as well as the lateral and medial septal nuclei in the frog brain (Eisthen and Polese, 2010). They also innervate the lateral amygdala (Scalia et al., 1991b). Contrary to the lack of an apparent AOB in the teleost fish, the AOB in the frog is quite evident and is located at the ventrolateral region of the OB. Anterograde HRP tracing experiments revealed that the AOB projection neurons project to the medial amygdala (Scalia et al., 1991b). While the projection from the MOB and AOB may converge in the amygdala, the medial amygdaloid nucleus may be connected exclusively to the AOB (Scalia et al., 1991b) (Figure 2B2).

### Reptiles

Whether a single OSN of reptiles projects unbranched axon to a single glomerulus has not been clearly described. The MOB of reptiles also exhibits a distinct layer structure in which the GL, EPL, MCL, and GCL are clearly visible, however, they may have a thicker IPL than mammalian OB (Kirillova and Lin, 1998; Pinato and Midtgaard, 2003; Kondoh et al., 2013). Mitral cells are distributed throughout the MCL in the turtle OB (Kirillova and Lin, 1998). The morphology of turtle mitral cells was determined by reconstruction after electrophysiological recordings and showed the existence of long secondary dendrites that reach almost half of the bulbar circumference (Mori et al., 1981). In the turtle and snake OB, a single mitral cell possesses more than one primary dendrite and therefore is capable of targeting multiple glomeruli (Mori et al., 1981; Iwahori et al., 1989) (Figure 2C1). However, mitral cells in the lizard OB have only one dendritic tuft in a glomerulus, which was shown with Golgi staining (Llahi et al., 1985) (Figure 2C1). While not yet experimentally concluded, the existence of tufted cells in the reptile OB is suggested due to the discernible segregation of the EPL and MCL. In fact, the mitral cells observed in the EPL of the lizard OB are described as displaced mitral cells (Llahi et al., 1985). Cells varying in soma size were identified in the EPL and MCL of the snake MOB (Kondoh et al., 2013), and the Japanese striped snake has two mitral cell types that are morphologically distinct based on the somata locations within the MCL (Iwahori et al., 1989). These support a concept suggestive of a transition between mitral cells and tufted cells.

In the snake brain, the axonal projections from the MOB terminate at the AON, olfactory tubercle (OT), the lateral cortex, and some amygdaloid nuclei (Halpern, 1976; Martinez-Garcia et al., 1991; Lohman and Smeets, 1993; Lanuza and Halpern, 1998; Eisthen and Polese, 2010; Ubeda-Banon et al., 2011) (Figure 2C2). Overall, the projection patterns of most reptilian MOB projection neurons are somewhat comparable to that of the rodent (see Figures 2C2, 4). However, in contrast to the mammalian OB projection neurons, some axons from the snake MOB enter the ipsilateral stria medullaris thalami, cross the midline in the habenular commissure, enter the contralateral stria medullaris thalami and terminate in the contralateral lateral pallium (Halpern, 1976; Lanuza and Halpern, 1998). The accessory olfactory system becomes more noticeable in reptiles compared to amphibians considering that the AOB is caudally located and clearly separated from the MOB (Martínez-Marcos and Halpern, 2009; Ubeda-Banon et al., 2011; Kondoh et al., 2013). The AOB mitral cells in the reptile give rise to more than one primary dendrite with multiple tufts in the GL (Llahi et al., 1985). The axons of AOB mitral cells follow along the accessory olfactory tract and project to three portions of the amygdala: the nucleus sphericus, medial amygdala, and nucleus of the accessory olfactory tract (Halpern, 1976; Martinez-Garcia et al., 1991; Lohman and Smeets, 1993; Lanuza and Halpern, 1998; Eisthen and Polese, 2010; Ubeda-Banon et al., 2011) (Figure 2C2). In some reptilian species including a type of lizard, the AOB projects to the bed nucleus of stria terminalis (BNST) (Martinez-Garcia et al., 1991).

### Evolutionary Morphological Changes of OB Projection Neurons

Different species have morphologically distinct MOB projection neurons. As described above, the mitral cells of teleost fish, amphibians, and reptiles, excluding zebrafish and lizards, typically possess multiple primary dendrites projecting to multiple glomeruli while only a single primary dendrite is formed in the mammalian mitral cells (Kosaka and Hama, 1982; Oka, 1983; Fujita et al., 1988; Satou, 1990; Dryer and Graziadei, 1994; Fuller et al., 2006). These results suggest that OB projection neurons have reduced the number of their primary dendrites from multiple to a single primary dendrite over the course of evolution. Interestingly, rodent MOB mitral cells form multiple primary dendrites with tufts in multiple glomeruli at the initial stage of development (Malun and Brunjes, 1996; Lin et al., 2000). Although it is reasonable to assume that having a single primary dendrite projecting to a single glomerulus assists in odor discrimination, it is still largely unknown how each mitral cell is able to “select” one dendrite during maturation. A recent study showed that spontaneous network activity among immature projection neurons in the neonatal OB is essential for the pruning of excess primary dendrites, but the OSN activity does not appear to be necessary in this process (Lin et al., 2000; Fujimoto et al., 2019). Comparing the development of OB projection neurons in different species would provide us with interesting insights into the molecular and cellular mechanisms underlying the selection of a single primary dendrite.

It is also noteworthy that clear secondary dendrites seem to be first apparent in the reptilian mitral cells, whereas the distinction between the primary and secondary dendrites in amphibian mitral cells is difficult to observe. From an evolutionary perspective, this information suggests that information processing in the OB became more intricate and complex as life began to shift from living in water to living on land. As discussed in the latter section, difference in the length of secondary dendrites is important for OB projection neurons to differentially respond to the odor inputs in the rodent OB. Formation of the two characteristic dendrites might be a stepping stone to generate the parallel processing pathways in the olfactory system.

## Similarities and Differences Between Mitral and Tufted Cells in the Rodent OB

Henceforth, we will focus on the subpopulation of MOB projection neurons reported from the rodent olfactory system. The projection neurons in the rodent OB are essentially classified into two types; mitral cells, named after their shape resembling that of a bishop’s miter, and tufted cells, named by Ramon y Cajal (1911) and Figueres-Onate et al. (2014). An increasing number of studies report that the two cell types exhibit different properties in response to odor stimuli, and dendrite/axonal projection patterns, suggesting that mitral and tufted cells process different aspects of olfactory information as described below.

### Morphological Properties

In the rodent OB, the projection neurons located in the MCL are defined as mitral cells, and others found in the EPL and GL are deemed tufted cells (Ramon y Cajal, 1911; Shepherd et al., 2004; Greer et al., 2008; Ennis et al., 2015). However, based on the relative depth of somata location in the EPL and GL, tufted cells are further subclassified into the external, middle, and internal tufted cells, also known as superficial, intermediate, and deep tufted cells, respectively (Ramon y Cajal, 1911; Macrides and Schneider, 1982; Orona et al., 1984; Shepherd et al., 2004). The internal tufted cells are sometimes identified as displaced mitral cells because of their proximity to the MCL (Mori et al., 1983; Kishi et al., 1984; Shepherd et al., 2004). The size of mitral cell somata (20–25 μm) is typically larger than that of tufted cells (10–20 μm) (Pinching and Powell, 1971a). Both mature mitral and tufted cells have a single primary dendrite with a tuft at the tip residing in a single glomerulus where they receive excitatory input from OSN axons (Ramon y Cajal, 1911; Mori et al., 1983). Mitral and tufted cells also form dendrodendritic synapses with inhibitory periglomerular interneurons in the GL (Pinching and Powell, 1971b).

**FIGURE 3:**
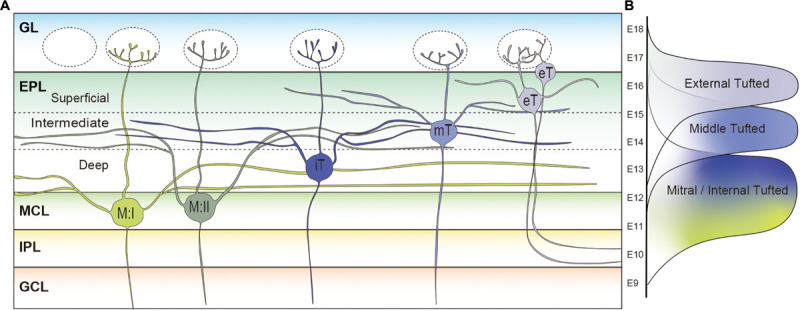
Subpopulations of projection neurons in rodent main olfactory bulb. **(A)** Morphological differences of projection neurons in the rodent olfactory bulb (OB). Type I mitral cells (M:I) extend their secondary dendrites in the deepest sublayer of the external plexiform layer (dEPL) while those of type II mitral cells (M:II) project to the intermediate EPL (iEPL). Somata of internal tufted cells (iT) are found in the dEPL, and their secondary dendrites extend in the iEPL. Middle tufted cells are mostly located in the iEPL. Tufted cells located in the superficial EPL (sEPL) or glomerular layer (GL) are classified as external tufted cells (eT). The external tufted cells are further subclassified into cells with secondary dendrites projecting to the sEPL or cells absent of secondary dendrites. The deeper the location of projection neurons cell somata in the OB, the longer the secondary dendrites are. **(B)** Timings of MOB projection neuron generation in the developing mouse brain. Mitral cells are generated between E9 and E13 followed by the middle (E12–E16) and external (E13–E18) tufted cells.

In the rodent OB, different types of projection neurons possess different lengths of secondary dendrites. Mori et al. studied projection neurons in the rabbit OB, and showed that a single mitral cell possesses ∼15,000 μm of secondary dendrites, which is almost four times longer than that of middle tufted cells (∼4,000 μm) (Mori et al., 1983). Internal tufted cells have an intermediate length of secondary dendrites (∼12,000 μm) (Mori et al., 1983). Also in the mouse OB, it was shown that the total dendritic length of a mitral cell (∼15,000 μm) is much longer than that of a middle tufted cell (∼7,500 μm) (Igarashi et al., 2012). The tufted cells located in the most superficial EPL or GL are classified as the external tufted cells. Like the other mitral and tufted cells, the primary dendrites of external tufted cells are generally uni-glomerular, with a small subpopulation being di-glomerular (Ennis and Hayar, 2008). There are two distinct subpopulations of external tufted cells, and the most prominent dissimilarity between the populations is the presence or absence of secondary dendrites (Macrides and Schneider, 1982; Hayar et al., 2004a, b; Antal et al., 2006; Liu and Shipley, 2008; Hirata et al., 2019). The somata of external tufted cells with secondary dendrites are generally found in the deeper one-third of the GL, or in the EPL near the boundary with the GL. Morphometric analysis using 300 μm thick rat OB slices showed that the total length of secondary dendrites of external tufted cells in the slice was ∼1,200 μm (Antal et al., 2006). Although we cannot directly compare the results due to the differences in species and method, it is reasonable to assume that mitral cells possess the longest secondary dendrites while those of the external tufted cells are the shortest. Interestingly, the other group of external tufted cells whose somata are only found in the GL lack secondary dendrites (Macrides and Schneider, 1982; Hayar et al., 2004a, b; Antal et al., 2006; Liu and Shipley, 2008; Hirata et al., 2019). Based on the data acquired thus far, it can be asserted that the deeper the location of projection neurons cell somata in the OB, the longer the secondary dendrites are as shown in Figure 3A.

In addition to the differences in length, Figure 3A shows that mitral and tufted cells extend their secondary dendrites in different sublayers of the EPL. The secondary dendrites of mitral cells appear to remain restricted to the deepest portion of the EPL (dEPL), proximal to the MCL, while those of tufted cells extend in the most superficial portion of the EPL (sEPL), proximal to the GL (Macrides and Schneider, 1982; Mori et al., 1983; Orona et al., 1984; Imamura and Greer, 2015). Even among tufted cells, it has been shown that the external tufted cells extend secondary dendrites to the most outer region of the sEPL and the middle tufted cells to the inner region of the sEPL (Macrides and Schneider, 1982; Mori et al., 1983). Several studies defined an intermediate EPL (iEPL) between sEPL and dEPL, where the secondary dendrites of internal tufted cells are projected (Orona et al., 1984; Mouradian and Scott, 1988). As discussed in a later section, there are mitral cells that extend secondary dendrites to the iEPL or the inner sEPL (Orona et al., 1984). Therefore, the regions in the EPL occupied by secondary dendrites of mitral cells and tufted cells gradually shift from deep to superficial. Interestingly, granule cells are also subgrouped into at least three populations based on their dendritic extension patterns in the EPL: the type-I granule cell ramifies spiny dendrites at any depth of the EPL; dendrites of the type-II granule cell extend only in the deep EPL; and the type-III granule cell ramifies spiny dendrites predominantly in the superficial EPL (Schneider and Macrides, 1978; Mori et al., 1983; Orona et al., 1983; Merkle et al., 2007). A computational analysis indicated that lateral inhibition mediated by dendrodendritic synapses between secondary dendrites of projection neurons and granule cell dendrites in the EPL could spread through granule cells only in a mitral-mitral or tufted-tufted way, but not mitral-tufted (Cavarretta et al., 2018). However, it was also shown that individual granule cells could influence a large group of both mitral and tufted cells belonging to at least 15 glomerular modules (Arnson and Strowbridge, 2017).

The activity of OB projection neurons is also regulated by other interneurons, such as short-axon cells (Pressler and Strowbridge, 2006; Parrish-Aungst et al., 2007; Eyre et al., 2008; Eyre et al., 2009; Arenkiel et al., 2011; Burton, 2017; Burton et al., 2017). In the EPL, mitral and tufted cells form reciprocal and non-reciprocal connections with EPL-located interneurons (EPL-IN) expressing parvalbumin (PV), somatostatin (SST), vasoactive intestinal peptide (VIP), and/or Corticotropin-Releasing Hormone (CRH) (Toida et al., 1994; Lepousez et al., 2010; Huang et al., 2013; Matsuno et al., 2017). EPL-INs form broader patterns of connectivity with mitral and tufted cells than granule cells (Huang et al., 2016), which is consistent with the broader odor tuning of EPL-INs (Kato et al., 2013; Miyamichi et al., 2013). Interestingly, CRH-positive EPL-INs provide stronger inhibition onto tufted cells than mitral cells, and tufted cells exhibit more linearly additive responses to odor mixtures without EPL-IN inhibitions (Liu G. et al., 2019). On the other hand, SST-positive EPL-INs are located in the dEPL and extend dendrites specifically into the dEPL (Lepousez et al., 2010). Together with the results showing that lateral inhibition differs between mitral cells and tufted cells (Geramita et al., 2016; Geramita and Urban, 2017; Matsuno et al., 2017), these results suggest that mitral and tufted cell activities are regulated mostly by different inhibitory circuits.

Axons of OB projection neurons ramify both within the OB and in the olfactory cortex (Kishi et al., 1984; Ojima et al., 1984; Orona et al., 1984; Igarashi et al., 2012). Within the OB, the axon collaterals terminate predominantly in the GCL to form asymmetric synapses on somata and dendrites of granule cells and short axon cells (Price and Powell, 1970a, b; Eyre et al., 2008). In the rabbit OB, the collaterals of mitral cells were distributed widely from the deep portion to the most superficial portion of the GCL (Kishi et al., 1984). The collaterals of internal tufted cells tended to be distributed more superficially in the GCL than mitral cells while those of middle tufted cells were distributed in the most superficial GCL (Kishi et al., 1984). It was also reported that the collaterals of external tufted cells run through the IPL to connect lateral and medial sides of the odor map (Liu and Shipley, 1994; Belluscio et al., 2002; Lodovichi et al., 2003). Since granule cells that project their dendrites to the superficial and deep EPL tend to localize in the superficial and deep GCL, respectively (Orona et al., 1983; Imamura et al., 2006), different types of OB projection neurons seem to construct distinct neuronal microcircuits within the OB.

**FIGURE 4:**
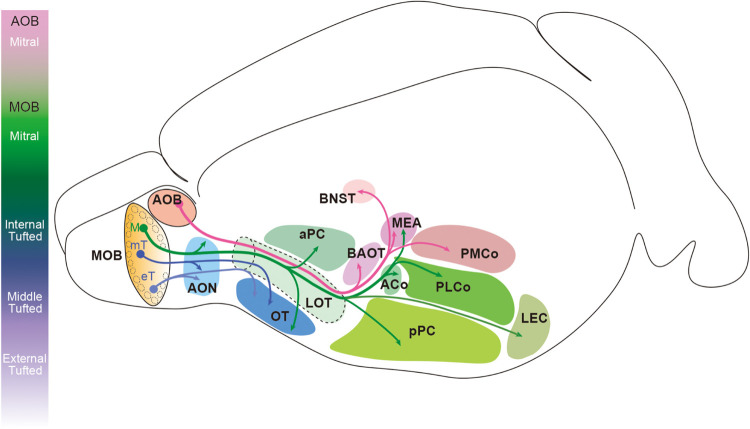
Innervation patterns of olfactory bulb projection neurons in the rodent brain. Mitral cells in the accessory olfactory bulb (AOB) project their axons to the bed nucleus of the accessory olfactory tract (BAOT), the bed nucleus of stria terminalis (BNST), the medial amygdaloid nucleus (MEA), and the posteromedial cortical amygdaloid nucleus (PMCo). Mitral cells (M) in the main olfactory bulb (MOB) innervate the anterior olfactory nucleus (AON), the anterior and posterior piriform cortex (aPC and pPC), the olfactory tubercle (OT), the lateral entorhinal cortex (LEC), the MEA, and the anterior and posterolateral cortical amygdaloid nucleus (ACo and PLCo). However, the axons of tufted cells (mT and eT) project only to the anterior portion of the olfactory cortex including the pars externa of the AON and the anterolateral OT.

Projection neuron axons extend from the ventrolateral side of the OB and form the LOT before innervating the olfactory cortex (Kishi et al., 1984; Yamatani et al., 2004; Walz et al., 2006; Igarashi et al., 2012). AOB mitral cell axons pass through the deepest layer of the LOT, and the axons of the MOB mitral and tufted cells are found in the intermediate and superficial layers, respectively (Inaki et al., 2004; Yamatani et al., 2004). The location of the axons assists in preserving the topographical organization of the olfactory information as it extends from the OB to the olfactory cortex. Target regions of the AOB mitral cells and MOB mitral and tufted cells rarely overlap (Figure 4). The AOB mitral cells transmit the information from the vomeronasal organ to the bed nucleus of the accessory olfactory tract (BAOT), the BNST, the medial amygdaloid nucleus (MEA), and the posteromedial cortical amygdaloid nucleus (PMCo) (Scalia and Winans, 1975; Davis et al., 1978; Yoshihara et al., 1999; von Campenhausen and Mori, 2000). The MOB mitral and tufted cells innervate the olfactory cortex consisting of the AON, the anterior and posterior piriform cortex (aPC and pPC), the OT, the lateral entorhinal cortex (LEC), the MEA, and the anterior and posterolateral cortical amygdaloid nucleus (ACo and PLCo) (Scalia and Winans, 1975; Haberly and Price, 1977; Yoshihara et al., 1999; Miyamichi et al., 2010; Ghosh et al., 2011; Sosulski et al., 2011; Hintiryan et al., 2012; Igarashi et al., 2012; Hirata et al., 2019). Within the MEA, AOB mitral cell axons terminate in the deep region, and the MOB mitral cell axons are found in the superficial layer without overlap (Kang et al., 2009). Several studies showed that a single mitral cell innervates the entire olfactory cortex while a tufted cell projects only to part of the AON and OT (Nagayama et al., 2010; Igarashi et al., 2012; Hirata et al., 2019). Within the OT, middle tufted cells project to the lateral portion, whereas the medial portion is preferentially innervated by mitral cells (Igarashi et al., 2012). Although it was previously undetermined if external tufted cells project their axons outside the OB (Schoenfeld et al., 1985; Tobin et al., 2010; Lukas et al., 2019), Hirata et al. (2019) recently showed that at least a subpopulation of external tufted cells do target the anterolateral edge of the OT as well as the pars externa of the AON.

### Physiological Properties

Odor responses of mitral and tufted cells are regulated by their intrinsic physiological properties and the intraglomerular and interglomerular circuitry (Aungst et al., 2003; Shao et al., 2012, 2013; Adam et al., 2014; Fukunaga et al., 2014). Many studies have reported the dissimilarity in odor responses between mitral and tufted cells. First, a distinct combination of odorants excites or suppresses the output of mitral and tufted cells. Interestingly, a specific odor can inhibit mitral cell excitation, while the external tufted cells show a distinct lack of the odor-specific suppression which is believed to be shaped by interglomerular inhibition (Adam et al., 2014; Kollo et al., 2014; Banerjee et al., 2015; Economo et al., 2016). Molecular receptive range (MRR) is defined as the odor spectrum that activated by a given odorant receptor, glomerulus, or a mitral or tufted cell, and is important for odor discrimination. It was reported that middle tufted cells have a broader MRR than mitral cells (Nagayama et al., 2004; Adam et al., 2014). Similarly, even among the projection neurons targeting the same glomerulus, the deeper the somata location, the narrower the MRRs (Kikuta et al., 2013). Since the MRRs of OB projection neurons are largely regulated by lateral inhibition mediated by the dendrodendritic synapses formed between mitral/tufted cells and granule cells (Yokoi et al., 1995; Tan et al., 2010; Geramita et al., 2016), the length of the secondary dendrites may be a critical determinant of the MRR.

It has been suggested that not only the MRRs but also the temporal components of the projection neuron activity contributes to odor identification (Schaefer and Margrie, 2007; Uchida et al., 2014; Wilson et al., 2017; Blazing and Franks, 2020). There are notable differences between mitral and tufted cells in temporal activation patterns after odor stimulation. Tufted cells can respond to lower concentrations (∼10 times lower than mitral cells) of odor stimuli with a higher frequency (>100 Hz), whereas the typical firing rate of mitral cells is less than 100 Hz (Nagayama et al., 2004; Igarashi et al., 2012; Kikuta et al., 2013; Adam et al., 2014). *In vitro* studies suggested that the greater excitability of tufted cells is caused by stronger afferent excitation, greater intrinsic excitability, and less inhibitory tone (Schneider and Scott, 1983; Burton and Urban, 2014; Arnson and Strowbridge, 2017; Geramita and Urban, 2017). On the other hand, mitral cells respond to strong OSN stimulation with sustained firing, or persistent discharge, that continues after odor stimulation (Adachi et al., 2005; Matsumoto et al., 2009; Geramita and Urban, 2017; Vaaga and Westbrook, 2017). The timing of firing onset in reference to the respiratory cycle is also different between mitral and tufted cells. Tufted cell spiking is phase-locked to OSN stimulation without sustained firing and starts during the middle of the inhalation phase (early-onset), while mitral cells respond with later-onset during the transition phase from inhalation to exhalation in anesthetized freely breathing rodents (Fukunaga et al., 2012; Igarashi et al., 2012). However, in an artificial inhalation paradigm, superficial, middle, and deep projection neurons were not reliably distinguished based on the timing of their inhalation-evoked activity (Diaz-Quesada et al., 2018; Short and Wachowiak, 2019).

External tufted cells receive direct OSN input and provide feedforward excitation to other neurons in the GL including periglomerular and short-axon cells and therefore are involved in interglomerular suppression of other OB projection neurons (Aungst et al., 2003; Hayar et al., 2004a; Whitesell et al., 2013; Liu and Liu, 2018). In addition, as described in the previous section, at least a subset of external tufted cells target their axons to the anterolateral edge of the OT and the pars externa of the AON (Hirata et al., 2019), suggesting that they contribute to parallel pathways of the olfactory system. Focusing on intrinsic physiological properties, the external tufted cells inherently generate rhythmic theta bursts (1–10 Hz) of action potentials and respond optimally to rhythmic, sniffing-related input (Hayar et al., 2004b; Liu and Shipley, 2008). On the other hand, mitral cells have biphasic membrane potentials that control the responsivity to OSN stimuli (Heyward et al., 2001; Kollo et al., 2014). As suggested from the differences in intrinsic properties, responses to odor stimuli of external tufted cells are distinct from mitral cells (Vaaga and Westbrook, 2016, 2017). Moreover, cholecystokinin (CCK) is a neuropeptide that is known to express strongly in a subset of the external tufted cells (Seroogy et al., 1985; Liu and Shipley, 1994; Gutierrez-Mecinas et al., 2005; Baltanas et al., 2011), although *in situ* hybridization analysis and recent immunohistochemical studies indicate a weak CCK expression also in mitral cells (Ingram et al., 1989; Hirata et al., 2019). Optical imaging of different mouse OB projection neurons showed that CCK-positive external tufted cells exhibited a shorter range of odor response latencies and durations than mitral cells and other external tufted cell populations (Short and Wachowiak, 2019). Thus, external tufted cells likely transmit the olfactory information to specific regions in the olfactory cortex with unique temporal patterns. On the other hand, vasopressin, a neuropeptide, is predominantly expressed by external tufted cells with secondary dendrites and some middle tufted cells, but not by mitral cells, in the rodent OB (Tobin et al., 2010; Lukas et al., 2019). It is proposed that the vasopressin-positive external tufted cells are involved in mechanisms of social recognition via the odor signatures (Dluzen et al., 1998a, b; Tobin et al., 2010; Wacker et al., 2011). However, recent studies showed that OSN stimulation primarily caused strong inhibition of the vasopressin-positive external tufted cells, suggesting that OSN axons do not directly activate them (Lukas et al., 2019). Whether the vasopressin-positive external tufted cells form a distinct neural circuit/pathway from the other external tufted cells is needed to be further elucidated.

The various types of oscillations of local field potential (LFP) are produced in the OB after odor stimulation, and they are associated with odor perception, discrimination, and learning (Martin et al., 2004; Beshel et al., 2007; Kay et al., 2009; Lepousez and Lledo, 2013; Martin and Ravel, 2014; Li et al., 2015; Liu P. et al., 2020; Losacco et al., 2020). A previous report showed that the local circuits produce fast-frequency (65–100 Hz) and slow-frequency (35–65 Hz) gamma oscillations of LFP in the OB (Kay, 2003). Several reports suggested that the early-onset fast gamma-oscillations and later-onset slow gamma oscillations are generated mainly by tufted cell and mitral cell subsystems, respectively (Manabe and Mori, 2013; Frederick et al., 2016). On the other hand, it was shown that glomerular networks coordinate theta oscillations (2–12 Hz) (Hayar et al., 2004b; Fukunaga et al., 2014). These findings imply that synchronized oscillatory activity at different frequency may be a key mechanism for OB projection neurons to process the different aspects of odor information in parallel.

### Heterogeneity of Mitral Cells

Since the MCL is only about the size of one cell body in thickness, heterogeneity of mitral cells has not been deeply investigated. However, several lines of evidence indicate that mitral cells do consist of heterogeneous subpopulations. Although, as noted above, mitral cells usually extend their secondary dendrites in the dEPL (Mori et al., 1983; Orona et al., 1984), some mitral cells extend their secondary dendrites in the iEPL in the rat OB, even though their somata lay in the MCL (Orona et al., 1984; Mouradian and Scott, 1988). Orona et al. (1984) classified the former mitral cells as Type I and the latter as Type II mitral cells (Figure 3A). Interestingly, the total secondary dendrite length of Type II mitral cells was longer than that of the middle tufted cell but shorter than Type I mitral cells (Orona et al., 1984). It remains to be seen whether there is a difference in axonal projection patterns between Type I and Type II mitral cells.

Mitral cells may also be subclassified based on their location along different planes of the OB. For example, mitral cells located at different regions along the dorsomedial-ventrolateral axis in the MCL tend to exhibit different projection patterns toward the OT, cortical amygdala, and MEA (Haberly and Price, 1977; Scott et al., 1980; Miyamichi et al., 2010; Imamura et al., 2011; Inokuchi et al., 2017). Retrograde labeling of OB projection neurons from the olfactory cortex revealed that the cortical amygdala (ACo and PLCo) and OT receive afferent projections preferentially from mitral cells in the dorsomedial and ventrolateral MCL, respectively (Haberly and Price, 1977; Scott et al., 1980; Miyamichi et al., 2010; Imamura et al., 2011). MOB mitral cells that project to the MEA are locally found at the ventral region of the OB and mediate odor-induced attractive social responses (Lin et al., 2007; Thompson et al., 2012; Inokuchi et al., 2017; Lemons et al., 2017).

In contrast to the studies reporting differences between mitral and tufted cells, only a few studies have suggested heterogeneous physiological and molecular properties among mitral cells. It has been shown that the α3 subunit of the GABA_A_ receptor, as well as a subunit of voltage-gated potassium channel (Kv1.2), are expressed by subsets of mitral cells (Panzanelli et al., 2005; Padmanabhan and Urban, 2010). In addition, a subunit of hyperpolarization-activated cyclic nucleotide-gated channel, HCN2, was expressed in glomeruli in a mosaic pattern (Angelo and Margrie, 2011; Angelo et al., 2012). These studies also reported on the diversity of intrinsic biophysical properties among mitral cells, such as firing frequency and the *I*_h_ sag current, which are supposedly reflective of varying Kv1.2 and HCN2 expression levels (Padmanabhan and Urban, 2010; Angelo et al., 2012). Moreover, these differences in molecular and biophysical properties may endow mitral cells with different odor response properties (Dhawale et al., 2010; Kikuta et al., 2013).

## Implications of Parallel Pathways for Olfactory Processing

Increasing evidence has suggested that mitral and tufted cells differentially transmit olfactory information to the olfactory cortex even when they receive OSN inputs within the same glomerulus. It is noteworthy that the piriform cortex is innervated only by mitral cell axons, and the piriform cortex is one of the major brain regions that serves a critical role in odor encoding, odor identification across different odor concentrations, odor learning, and discrimination and perception of complex odor mixtures (Wilson, 2000; Barnes et al., 2008; Stettler and Axel, 2009; Chapuis and Wilson, 2011; Choi et al., 2011; Haddad et al., 2013; Bolding and Franks, 2017, 2018; Iurilli and Datta, 2017; Roland et al., 2017; Meissner-Bernard et al., 2019); see also reviews in Wilson and Sullivan (2011), Bekkers and Suzuki (2013), Blazing and Franks (2020). The narrower MRR of mitral cells is likely advantageous in the process of accurate odor encoding. However, it is also suggested that mitral cells in the rat MOB do not receive lateral inhibition broadly from surrounding glomeruli via interneurons, but rather receive lateral inhibition from only a small number of spatially distributed glomeruli (Fantana et al., 2008; Shmuel et al., 2019). Moreover, individual odors activate ensembles of spatially distributed neurons in the piriform cortex that lack apparent topographical organization with respect to the odor map (Stettler and Axel, 2009; Miyamichi et al., 2010; Ghosh et al., 2011; Sosulski et al., 2011; Igarashi et al., 2012). Instead, it is proposed that neurons in the piriform cortex stochastically sample glomeruli to generate a systematic population-level representation to identify the odors (Schaffer et al., 2018; Pashkovski et al., 2020). Thus, the patterns of connectivity from mitral cells to neurons in the piriform cortex should be elucidated at the level of synapses, which may identify novel mitral cell subpopulations and pathways.

The tufted cell pathway has a more rapid activation with lower odor concentration compared to mitral cells. This implies that tufted cells may transmit information from the glomeruli to the olfactory cortex with less spatial and temporal modification, and, therefore, may be involved in the olfactory functions in which speed is required for efficient processing. Morphological analyses showed that tufted cells, including the external tufted cells, project to part of the AON and the lateral OT (Nagayama et al., 2010; Igarashi et al., 2012; Hirata et al., 2019). It has been widely argued that the OT is involved in reward and motivational aspects of odor information processing, primarily due to the fact that the OT is a component of the ventral striatum that connects with the reward system, including the ventral tegmental area (Ikemoto, 2007; Wesson and Wilson, 2011; Gadziola et al., 2015; Yamaguchi, 2017; Zhang et al., 2017). It is particularly noteworthy that an odor associated with punishment activates the lateral domain of the OT and induces aversive behavior (Murata et al., 2015; Yamaguchi, 2017). The neural pathway originating from tufted cells may be necessary to escape quickly from these aversive odor sources. In contrast, an odor associated with reward activates the anteromedial domain of the OT and induces attractive behavior (Murata et al., 2015; Yamaguchi, 2017; Zhang et al., 2017), which may be mediated via the mitral cell pathway. Neurons in the AON that receive inputs from most of the MOB projection neurons decussate to the contralateral OB (Schoenfeld et al., 1985; Scott et al., 1985; Illig and Eudy, 2008; Yan et al., 2008). A previous report showed that AON neurons exhibit respiration phase-locked firing pattern to ipsi-nostril stimulation, and this activity is attenuated by contra-nostril stimulation, indicating that the AON is involved in the function of odor source localization (Kikuta et al., 2010; Liu A. et al., 2020). Since a subset of external tufted cells targets the anterolateral edge of the OT as well as the pars externa of the AON (Hirata et al., 2019), this external tufted cell subset may play a critical role in the behaviors induced by reward-related odors and odor source localization.

Another well-known feature of the external tufted cells is to link the isofunctional glomeruli within the OB (Schoenfeld et al., 1985; Liu and Shipley, 1994). It has been known that each rodent OB has two mirror-image OR maps, one in the lateral side and the other in the medial side, within which the two glomeruli representing a particular OR are mapped symmetrically (Nagao et al., 2000). The axon collaterals of an external tufted cell run through the IPL and terminate beneath the mitral cells at the corresponding region of the other side of the two maps (Belluscio et al., 2002; Lodovichi et al., 2003). The axons synapse onto the dendrites of granule cells within the IPL and therefore inhibit the surrounding mitral and tufted cells, which results in mutual inhibition between the lateral and medial maps (Belluscio et al., 2002). Although the functional roles of each map in odor information processing are unknown, a few differences in odor response patterns between glomeruli in these maps have been reported (Zhou and Belluscio, 2008, 2012; Baker et al., 2019; Sato et al., 2020). The functional difference of two maps may be elucidated through further study on the external tufted cells.

## Generation of Different Types of OB Projection Neurons

The strategy to assign neurons having different birthdates with different properties is widely used in the brain to generate a variety of neuronal subtypes useful for processing complex information. In the developing retina, all types of cells, including Müller glia, are generated from a single pool of progenitors (Turner and Cepko, 1987; Bassett and Wallace, 2012). The fate of the precursors is largely determined by the timing of neurogenesis, namely that the first neurons born are the RGCs followed by cone photoreceptors, horizontal cells, amacrine cells, rod photoreceptors, and bipolar cells (Bassett and Wallace, 2012). Müller glia are the last of the cells to emerge in the retina. Even among the amacrine cells, it is suggested that the birthdates specify their destinations and subtype identities (Voinescu et al., 2009). The cerebral cortex is made up of six layers, and each layer contains pyramidal projection neurons that possess distinct dendritic morphologies and axonal target regions as well as different molecular expression profiles (Molyneaux et al., 2007; Kwan et al., 2012; Greig et al., 2013; Narayanan et al., 2017; Gerfen et al., 2018). The mouse cortical pyramidal neurons are generated between E11 and E18, and the neurons with different birthdates migrate toward distinct layers with an inside-out manner (Molyneaux et al., 2007; Kwan et al., 2012; Greig et al., 2013).

Similarly, the timing of neurogenesis is a major contributor to producing diversity in the OB projection neurons. The earliest generated projection neurons in the mouse OB are the AOB mitral cells that emerge around embryonic day (E) 9 and 10 (Hinds, 1968; Imamura and Greer, 2015; Hirata et al., 2019). A recent study indicated that the AOB and MOB projection neurons are generated from different progenitor cells whereas a single progenitor cell can give rise to both MOB mitral and tufted cells in the developing mouse OB (Sanchez-Guardado and Lois, 2019). Nevertheless, MOB mitral and tufted cells are generated at different time points; mitral cells are generated between E9 and E13 having a peak at E11, while middle and external tufted cells are born during a later period between E12 and E18 (Hinds, 1968; Blanchart et al., 2006; Imamura et al., 2011; Hirata et al., 2019) (Figure 3B). Thymidine analog labeling and genetic tracing experiments have shown that middle tufted cells are generated earlier than external tufted cells (Hinds, 1968; Winpenny et al., 2011; Hirata et al., 2019). Therefore, similar to cortical pyramidal neurons, projection neurons in different layers are also generated at different time points with an inside-out manner in the MOB.

Using the thymidine analog labeling method, we also showed that mitral cells generated at E9 or E10 (early-generated mitral cells) were preferentially localized to the dorsomedial MCL, while mitral cells generated at E12 or E13 (late-generated mitral cells) were predominantly located in ventrolateral MCL in the mouse OB (Imamura et al., 2011). Later, we further revealed that early- and late-generated mitral cells extend their secondary dendrites in the dEPL and iEPL, respectively, indicating that late-generated mitral cells can be classified as the previously identified Type II mitral cells (Orona et al., 1984; Imamura and Greer, 2015). These results strongly suggest that neuronal birthdate is a significant contributor in the generation of morphological differences, not only between mitral and tufted cells, but also among subpopulations of mitral cells. Based on mitral cell location contributing to the projection pattern of target structures in the olfactory cortex, neuronal birthdate may also be considered an implication of a cells function. However, a critical next step is to determine whether there are differences in physiological and/or molecular properties between early- and late-generated mitral cells.

In summary, the olfactory system processes multiple aspects of olfactory information through parallel pathways. Similar to the visual system, the diversity of OB projection neurons provides the basis for the parallel pathways in the rodent OB, which has been established throughout the course of evolution. In the retina, distinct RGC types have been characterized by dendritic and axonal arborization patterns as well as physiological parameters. More recently, molecular expression patterns, including transcription factors, have been used to examine the diversity of RGCs (Sanes and Masland, 2015; Baden et al., 2016; Rheaume et al., 2018). In this review, we have summarized the morphological and physiological diversities of OB projection neurons. Although differing molecular expression profiles of OB projection neurons in the rodent OB have yet to be identified, the timing of neurogenesis seems to regulate the generation of different projection neuron subpopulations. Thus far, a large number of transcription factors expressed in developing projection neurons in the rodent OB have been reported (Winpenny et al., 2011; Imamura and Greer, 2013), and we and others showed that each transcription factor appears in the developing OB with a distinct spatiotemporal pattern (Williams et al., 2007; Campbell et al., 2011; Nguyen and Imamura, 2019). In addition, the results from large-scale analyses using omics approaches are available (Campbell et al., 2011; Kawasawa et al., 2016). A combination of cutting edge techniques including single-cell RNA-sequencing, tissue-clearing and whole-brain imaging, optical imaging, and electrophysiological recordings can now be used to reveal molecular, morphological, and physiological properties of OB projection neurons. The knowledge acquired by these techniques will further elucidate the functions and ramifications of each pathway.

## Author Contributions

FI, AI, and BL reviewed the literatures and wrote the review. All authors contributed to the article and approved the submitted version.

## Conflict of Interest

The authors declare that the research was conducted in the absence of any commercial or financial relationships that could be construed as a potential conflict of interest.
